# Linear Dextrin as Potential Insulin Delivery System: Effect of Degree of Polymerization on the Physicochemical Properties of Linear Dextrin–Insulin Inclusion Complexes

**DOI:** 10.3390/polym13234187

**Published:** 2021-11-30

**Authors:** Huifang Xie, Xin Ma, Wenbin Lin, Shiting Dong, Qiang Liu, Yi Chen, Qunyu Gao

**Affiliations:** 1 Carbohydrate Laboratory, School of Food Science and Engineering, South China University of Technology, Guangzhou 510640, China; xiehf0213@gmail.com (H.X.); dsting0412@163.com (S.D.); lq272511@163.com (Q.L.); 2 School of Computer Science and Technology, Tiangong University, Tianjin 300387, China; mxtjcn@126.com (X.M.); linwbinI996@163.com (W.L.); 3 School of Materials Science and Engineering, South China University of Technology, Guangzhou 510006, China; cy63532499@163.com

**Keywords:** LD–insulin inclusion complexes, in vitro release, fabrication, DP, insulin, encapsulation

## Abstract

In the current study, linear dextrin (LD) was prepared using waxy potato starch debranched with pullulanase, which has attracted immense interest in the food, pharmaceutical, and cosmetic industries as a versatile ingredient. Various LDs were separated on the basis of their differential solubility in aqueous/ethanol solutions of different volumetric ratios. Three LD products—LD Fabrications with 40% ethanol (F-40); LD Fabrications with 50% ethanol (F-50); and LD Fabrications with 60%, 70%, and 80% ethanol (F-M)—were obtained with an average degree of polymerization (DP) values of 31.44, 21.84, and 16.10, respectively. The results of Fourier transform infrared spectroscopy (FT-IR) analysis revealed that the reaction mainly involved hydrogen bonding and a hydrophobic interaction between LD and insulin in the process of inclusion complex formation. X-ray diffraction (XRD) results indicated that insulin was encapsulated in LD. The results of circular dichroism (CD) showed that the changes in the secondary structure of insulin were negligible during the release from the inclusion complexes. The order of encapsulation capacity is as follows: the complex composed of F-M and insulin (F-M-INS) > the complex composed of LD and insulin (LD-INS) > the complex composed of F-50 and insulin (F-50-INS) > and the complex composed of F-40 and insulin (F-40-INS). F-M-INS inclusion complexes showed a better effect on reducing the release of insulin in gastric juice and promoting the release of insulin in intestinal juice and blood plasma than LD-INS.

## 1. Introduction

Dextrin is a hydrolysate of starch (excluding monosaccharides and oligosaccharides) that is produced by the cleavage of glycosidic bonds under the action of heating, acid, or amylase. According to the different treatment methods and the degree of action, dextrin can be divided into yellow dextrin [1], maltodextrin [2], limit dextrin [3], and many other types. When considering the molecular structure of dextrin itself, dextrin can be simply divided into three types: linear dextrin, branched dextrin, and cyclodextrin. Different from amylose, linear dextrin is a linear polymer in the strict sense, so it is also called LD. It usually consists of a dozen to hundreds of glucose units with α −(1, 4) connected by glycosidic bonds. Similar to most polysaccharides, such as pullulan and dextran, linear dextrin can exist in a randomly wound conformation in an aqueous solution, but the unique element is that linear molecular chains will be transformed into α in solution due to their own stereochemical constraints, the α-helical structure thus appearing to be amphipathic [4,5]. LD, also known as short linear glucan, short-chain amylose, and short maltodextrin, is produced when amylopectin is hydrolyzed by pullulanase or isoamylase [6,7,8,9,10]. LD has wide applications in the food and pharmaceutical industries, such as starch nanoparticles [11,12], inclusion complexes [13,14], and hydrogels [15]. LD shows high biocompatibility and biodegradability [16,17], which gives LD-based inclusion the optimal DP values of LD for encapsulating insulin, making it an ideal carrier system with applications in different fields, including food, cosmetics, pharmaceuticals, and tissue engineering [18]. LD has been widely used to improve the delivery of bioactive components [19], peptide and protein drugs [20], antigens [21], and vaccines [22]. Numerous nanoparticle systems have been extensively developed to enhance the bioavailability of insulin, such as natural polymeric [23], solid lipid [24], synthetic polymeric [25], liposome [26], and inorganic nanoparticles [27]. Compared to other nanoparticles, natural polymeric nanoparticles have emerged as one of the most promising systems for improved insulin delivery. Notably, they exhibit many favorable properties, including improved safety, biocompatibility, storage stability, and physiological stability, compared to other nanoparticles [28,29].

Chang et al. developed a simple fractionation method using an edible alcohol solution to obtain LD fractions of different molecular weights. LD fractions were separated from hybrid LD on the basis of their differential solubility in aqueous/ethanol solutions of different ratios (1:1.5 and 1:3). The DP values of the obtained LD samples were 23.53, 20.04, 9.14, and 6.75 [16]. Ji et al. used LD as a carrier to prepare short-chain amylose–insulin inclusion complexes through a self-assembly method. The results showed that the inclusion complexes have a particle size between 60 and 200 nm, are uniformly dispersed, and are nearly spherical in shape; the highest entrapment rate and loading capacity of insulin are 53.9% and 2.5%, respectively [30]. Chang et al. [31] used octenyl succinic anhydride (OSA) to modify debranched starch (DBS) with a low degree of polymerization (6.75) and good water solubility to obtain an amphiphilic polymer. The OSA-DBS vesicles spontaneously formed small bubbles of 10~30 nm in the water system. The vesicles were relatively stable under different temperatures, pH, and ionic strength. Insulin serves as a model of hydrophilic functional components. The vesicles loaded with insulin showed a higher loading content (13.36%). These OSA-DBS vesicles have potential applications as advanced carriers in the health food and biomedicine industries. Currently, there are very few reports available on LD fractions with different DP values encapsulated with insulin, and the optimal DP values of LD for encapsulating insulin are not clear.

Therefore, in the current study, a simple, scalable, and green method for producing LD fractions of different molecular weights was used. The separated LD fractions (DP = 16, 22, 31) encapsulated with insulin were characterized and compared with encapsulation efficiency, loading capacity, interactions, bioactivity, and in vitro release to understand their physicochemical properties. The results of this paper may provide a valuable data basis for research on encapsulating mechanisms between insulin and linear dextrin with different degrees of polymerization.

## 2. Materials and Methods

### 2.1. Materials

Waxy potato starch (approximately 2% amylose and 98% amylopectin) was purchased from Holland AVEBE Company. Pullulanase (EC 3.2.1.41) (4461.6 NPUN/g) was supplied by Sigma-Aldrich Trading Co., Ltd. (Shanghai, China). Insulin was supplied by Guangzhou Les Biological Technology Co., Ltd. (Guangzhou, China). All the reagents used were of analytical grade.

### 2.2. Fractionation of Debranched Starch by Gradient Ethanol Precipitation

Fractionation of debranched starch by gradient ethanol precipitation was carried out according to the method of Ranran Chang et al. [16], with minor modifications. Waxy potato starch (10 g, db) was dispersed in 100 mL of phosphate buffer solution (0.1 M, pH 4.6) and then cooked in a boiling water bath with vigorous stirring for 40 min. The fully gelatinized starch was cooled to 58 °C and debranched by pullulanase (6692 NPUN) for 24 h. The hydrolysate was quickly centrifuged (3500 g, 3 min) to avoid retrogradation. The supernatant was heated at 100 °C for 15 min to fully inactivate the enzyme and then centrifuged (3500 g, 3 min) to remove the enzyme. Subsequently, a fixed quantity of absolute ethanol was added to the hydrolysate solution quickly until the ethanol concentration was up to 90%. Linear dextrin was obtained; after being fully stirred and deposited at 4 °C for 24 h, centrifuged at 5000 r/min at 4 °C for 10 min, the supernatant was removed and freeze dried.

The fractionation of LD was conducted by gradient ethanol precipitation based on previous methods with minor modifications [32]. LD solution (W/W) 1% was dissolved under 100 °C and then cooled to room temperature. The LD solution was precipitated by slowly adding dehydrated ethanol under continuous stirring until reaching the final ethanol concentration of 40% (*v*/*v*) and kept at 4 °C for 24 h. Then, it was centrifuged at 4500 rpm for 5 min to obtain the precipitated fraction, which was defined as LD subfraction F-40. Dehydrated ethanol was further added slowly to the supernatant to reach a final ethanol concentration of 50% (*v*/*v*) and kept at 4 °C for 24 h. The precipitates (F-50) were obtained by centrifugation. A further increase in the ethanol concentration to 80% led to subfraction F-M.

### 2.3. Preparation of LD-INS, F-40-INS, F-50-INS, and F-M-INS Inclusion Complexes

In total, 1.0 (g, wb) linear dextrin and fractional linear dextrin components, namely LD, F-40, F-50, and F-M, were dispersed in 100 mL deionized water (1.0%, *w*/*v*). They were completely gelatinized in a boiling water bath and then lowered to room temperature. The insulin solution (10 mg insulin dissolved in 20 mL 0.01 mol/L HCl) was added to the above solution dropwise, magnetically stirred at 1000 r/min for 1 h to make it fully react, and then kept at 4 °C for 48 h. Then the solution was centrifuged to obtain the precipitations (the linear dextrin–insulin complexes), namely, LD-INS, F-40-INS, F-50-INS, and F-M-INS.

### 2.4. GPC Analysis

Standard curve: eight kinds of dextran standard products with different molecular weight (5200, 11,600, 23,800, 48,600, 148,000, 273,000, 410,000, and 668,000 Da) within the order of magnitude of 1 × 10^3^~1 × 10^7^ Da were dissolved into 2 mg/mL dextran standard solution in mobile-phase, low-concentration potassium dihydrogen phosphate aqueous solution. After filtration in a 0.45 μm water system, the analysis was carried out using a high-performance gel penetration chromatography (GPC) system. Breeze data processing software was used to process the data from the relative molecular weight of the dextran standard goods logarithmic (log Mw) on the elution volume (V) to establish the regression equation, namely the standard curve of the sample. A high-performance liquid chromatography pump (1525, Thermo, Waltham, MA, USA) and automatic sampler (717Plus, Thermo, Waltham, MA, USA) were used.

Determination of molecular weight: the samples were dissolved in the mobile phase until the final concentration reached 2~3 mg/mL, filtrated with 0.45 μm of water membrane. Sample quantity was 20 μL, and running time was 35 min. Breeze data processing software was used to calculate the distribution of the molecular weight of the samples.

The chromatographic conditions were as follows: the mobile phase was 0.02 mol/L KH2PO4 buffer solution; the gel column was in series using Ultra hydrogel 1000 (7.8300 mm) and Ultra hydrogel 500 (7.8300 mm); the flow rate was 0.8 mL/min; the Thermo 2414 differential detector was used; the column temperature was 35 °C.

### 2.5. Entrapment Efficiency (EE) and Loading Capacity (LC)

EE and LC were measured using a method reported previously with minor modifications [12]. Briefly, freshly prepared suspension was pipetted into the ultrafiltration tube (molecular weight cut off at 100 g/mol), followed by centrifugation at 10,000× *g* for 30 min at 4 °C using the high-speed refrigerated centrifuge machine (3–30 KS, Sigma, Berlin, Germany). The insulin content in the filtrate was determined using the previously reported HPLC method with small modifications [33]. Chromatographic analysis was carried out with an HPLC system equipped with a Thermoquest Spectra System P 1500 isocratic pump, spectra system UV 6000 LP photodiode array detection, SCM 1000 vacuum membrane degasser, and Chromquest software. Separation was achieved by using an Ace C18 column (5 mm, 4.6 250 mm i.d.; Merck) at a flow rate of 1 mL/min with acetonitrile 0.2 M Na_2_SO_4_ buffer solution (adjusted to pH 2.4 with H_3_PO_4_), 25:75 (*v*/*v*), as mobile phase. The mobile phase was freshly prepared every day. The mobile phase was premixed, filtered through a 0.45 μm membrane filter to remove the particulate matter, and degassed by sonication before use. DAD (190–600 nm) scanning was previously carried out to select the optimal absorbance wavelength. The sensitivity of the detector was set at 0.01 AUFS. The detection was performed at 206 nm, and the injection volume was 20 μL. Before injecting solutions, the column was equilibrated for at least 20 min with the mobile phase flowing through the system.

The EE and LC were determined using the following equation:

EE (%) = (TI − IS)/(TI) × 100(1)

LC (%) = (TI − IS)/(WN) × 100(2)

where TI is the total amount of insulin, IS is the amount of insulin in the supernatant, and WN is the total weight of the nanoinclusion complexes.

### 2.6. Determination of Fluorescence Spectra

An F-7000 fluorescence spectrophotometer (5J1-0004, Hitachi, Japan) was used to determine the interactions between linear dextrin and insulin according to the principle that fluorescence intensity changes with linear dextrin combined with insulin. LD, F-40, F-50, and F-M (1.0 g) were dissolved in 100 mL deionized water (1.0%, *w/v*), placed in a boiling water bath to be fully gelatinized, and cooled to room temperature. Insulin (10 mg) was dissolved in 20 mL 0.01 mol/L HCl solution. The insulin solution was added to the above solution dropwise and stirred at 1000 r/min for 1 h. The fluorescence spectrum of the sample was motivated at 260 nm, the scanning wavelength range was 280–500 nm, and the width of the excitation and emission slit were both 5.0 nm [34].

### 2.7. FT-IR Analysis

Samples (1~2 mg) and dry KBr (100~200 mg) were fully mixed in a high-speed mill and put into the pressure medium voltage to prepare tablets approximately 1 mm thick. Then, the sample was analyzed in FT-IR (Vector 33, Bruker, Berlin, Germany). Test conditions: the range of scanning wavenumber was 4000~400 cm^−1^, and the resolution was 4 cm^−1^. The DTGS detector was used, and air was used as blank. An average of 32 scans provided the infrared spectrum of the sample [35].

### 2.8. X-ray Diffraction (XRD) Patterns of the Samples

The X-ray diffraction (XRD) patterns of samples were examined with a D8 (Bruker, Billerica, MA, USA). Before the analysis, the freeze-dried samples were laid flat on the plate. The diffractogram scan was run between 5° and 40° (2θ) at a rate of 0.05°/s, Cu Ka radiation at 40 kV and 20 mA [36].

### 2.9. In Vitro Insulin Release

The insulin release was measured in accordance with previous studies with some modifications [31]. In total, 5 mL LD-INS and F-M-INS solutions were added to the dialysis bags (intercept molecular weight of 10,000 g/mol). Then, the dialysis bags were put into 100 mL PBS (phosphate-buffered saline) (pH 1.2, 6.8, and 7.4) at 37 °C of the water bath by shaking. At a predetermined time, 200 μL of supernatant was withdrawn; then, 200 μL of fresh PBS was added to the whole system. The released insulin amount was monitored by HPLC.

### 2.10. CD Spectra

The changes in the secondary structure of insulin before and after release were determined by CD (circular dichroism). The determination method referred to by Li et al. [37] was used to detect the above insulin released from gastric juice. The original insulin solution was used as the control, the concentration of insulin was configured at 1 mg/mL, and the wavelength scanning range was 190–250 nm.

### 2.11. Statistical Analysis

The differences between the mean values of multiple groups were analyzed using SPSS 17.0 and Origin 9.0 for one-way analysis of variance (ANOVA) with Duncan’s multiple range tests. ANOVA data with a *p* < 0.05 were classified as statistically significant. Mean values were from triplicate experiments.

## 3. Results and Discussion

### 3.1. GPC Results

**Figure 1:**
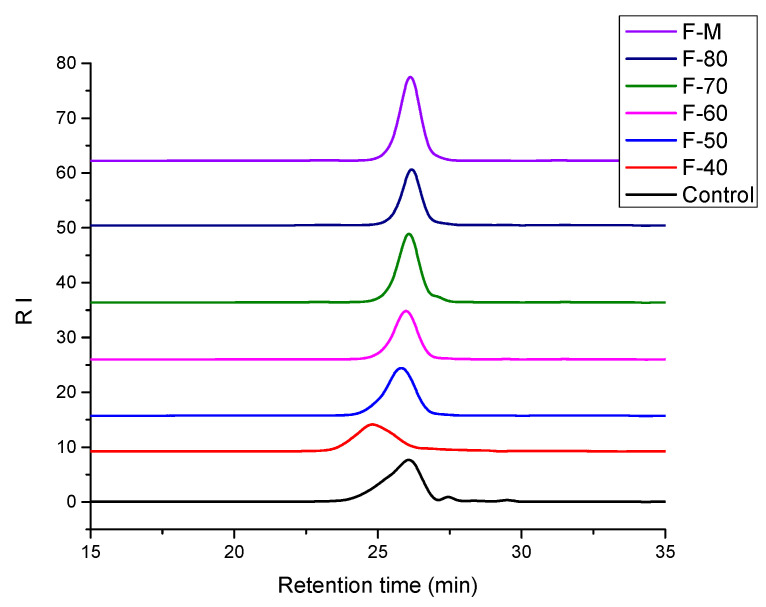
GPC pattern of LD fractions: LD, F-40, F-50, F-60, F-70, F-80, and F-M.

Figure 1 shows the gel penetration chromatograms of LD; F-40; F-50; LD Fabrications with 60% ethanol (F-60); LD Fabrications with 70% ethanol (F-70); LD Fabrications with 80% ethanol (F-80); and LD Fabrications with 60%, 70%, and 80% ethanol (F-M). Meanwhile, the relative molecular weight information of each gradient alcohol precipitation component of linear dextrin is also listed in Table 1. It is well known that both average molecular weight and molecular weight distribution are the two key characteristics that determine the properties of polymers, and the polydispersity index (PDI) is used as a measure of the breadth of the molecular weight distribution. PDI is defined as Mw/Mn, where Mw and Mn are the weight average and the number average molecular weight, respectively [38]. The narrower the relative molecular weight distribution of the samples, the closer the PDI value to 1. DP represents the degree of polymerization of the samples, and DP is defined as Mw/162.

From Table 1, we can see the molecular weight distribution of linear dextrin, F-40, F-50, and FM. The Mw of LD, F-40, F-50, and F-M was 3657, 5093, 3538, and 2609, respectively. The PDI values of LD, F-40, F-50, and F-M were 1.46, 1.19, 1.18, and 1.12, respectively. The PDI values of the fractionation (F-40, F-50, and F-M) of LD, which was conducted by gradient ethanol precipitation, were all lower than those of LD. This indicated that the molecular weight distribution range of fractionation (F-40, F-50, F-M) became narrow. Moreover, the higher the ethanol concentration, the smaller the average molecular weight. Consequently, we had three main fractionation components, F-40, F-50, and F-M, with an average DP of 31.44, 21.84, and 16.10, respectively.

### 3.2. Determination of Fluorescence Spectra

**Figure 2:**
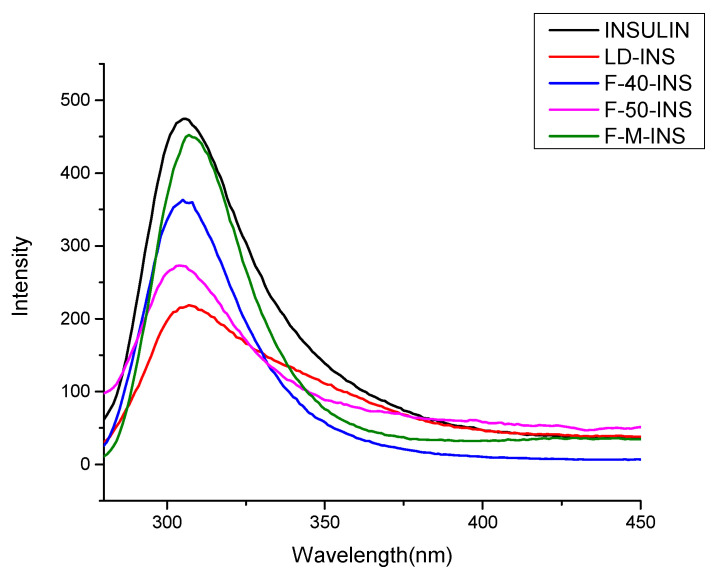
Fluorescence spectra of linear dextrin–insulin inclusion complexes: INSULIN, LD-INS, F-40-INS, F-50-INS, and F-M-INS.

Molecular interactions can make protein fluorescence characteristics change, so a fluorescence spectrum is useful for evaluating the interaction between the molecules of linear dextrin and insulin. The effect of linear dextrin of different DPs combined with insulin on fluorescent properties is shown in Figure 2. Insulin displayed a fluorescence emission peak at 307 nm under the excitation light of 260 nm.

The fluorescence intensity of the linear dextrin (not shown in the figure) is negligible because it was almost zero under the condition of the excitation light compared with the insulin. As seen in Figure 2, insulin fluorescence intensity decreased significantly as insulin was added into the linear dextrin. This indicates that the interactions between linear dextrin and insulin could cause insulin fluorescence quenching, which results in the decrease in fluorescence intensity. The fact that the fluorescence peak intensity of LD-INS inclusion complexes was lowest shows that the interactions between linear dextrin and insulin were the strongest, which indicates that insulin was in a more burial condition.

However, the fluorescence intensity of F-M-INS inclusion complexes was higher, which indicates that the interaction between F-M and insulin was weaker. F-M’s chain length is shorter, and the movement between the molecules is rapid, so the interaction between F-M and insulin was weaker. While F-50 belongs to the medium chain, the increase in the interaction between F-50 and insulin led to a reduction in the fluorescence intensity of F-50-INS inclusion complexes [39]. The middle-chain molecules were favorable to the formation of crystals, while the short-chain molecules could hinder the formation of crystals. Liu Wei’s report also found that the increase in the proportion of the middle and long chain in the debranched starch was favorable to the formation of the complex carrier network structure of the debranched starch and xanthan gum. The fluorescence peak intensity from low to high was as follows: LD-INS < F-50-INS < F-40-INS < F-M-INS < INSULIN. This indicates that interaction strength between linear dextrin and insulin from big to small was LD-INS > F-50-INS > F-40-INS > F-M-INS.

### 3.3. Entrapment Efficiency (EE) and Loading Capacity (LC)

The entrapment efficiency (EE) and loading capacity (LC) of LD-INS, F-40-INS, F-50-INS, and F-M-INS are shown in Table 2. It can be seen from Table 2 that the EE and LC from large to small are F-M-INS > LD-INS > F-50-INS > F-40-INS. With the chain length becoming shorter, the EE and LC of the samples increased gradually. The EE of LD-INS, F-40-INS, F-50-INS, and F-M-INS were 52.9%, 41.6%, 48.7%, and 68.2%, and the LC were 2.6%, 2.2%, 2.8%, and 3.4%, respectively. When the average DP was 31.44 (namely F-40), the EE and LC reached their lowest values; when the average DP was 16.10 (namely F-M), the EE and LC reached their maximum values, which were 68.2% and 3.4%, respectively.

### 3.4. FT-IR

**Figure 3:**
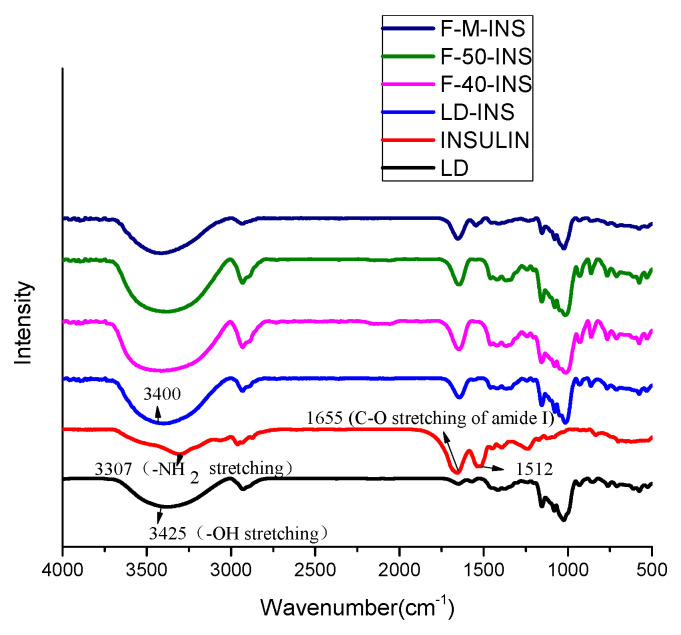
FT-IR spectra of LD, INSULIN, LD-INS, F-40-INS, F-50-INS, and F-M-INS inclusion complexes.

The FT-IR spectrum of LD, INSULIN, LD-INS, F-40-INS, F-50-INS, and F-M-INS are shown in Figure 3. The FTIR spectra of LD showed a peak at 3425 cm^−1^ (-OH stretching) [40], and insulin showed a peak at 3307 cm^−1^ (-NH2 stretching).

The shift of this band to 3400 cm^−1^ of the complex indicates that there was a hydrogen bond interaction between the -OH group of LD and the -NH2 group of insulin. The FT-IR spectra of insulin showed peaks of 1655 cm^−1^ (C-O stretching of amide I) and 1512 cm^−1^. When the inclusion complexes were formed, the peak positions of the amide I band and the amide II band did not change significantly, and the peak intensity gradually increased. Therefore, the results of the FT-IR spectrum indicate that there was an interaction between LD and insulin during the formation of the inclusion complexes, which mainly includes hydrogen bonding and hydrophobic interaction.

### 3.5. X-Diffraction Patterns of the Sample

**Figure 4:**
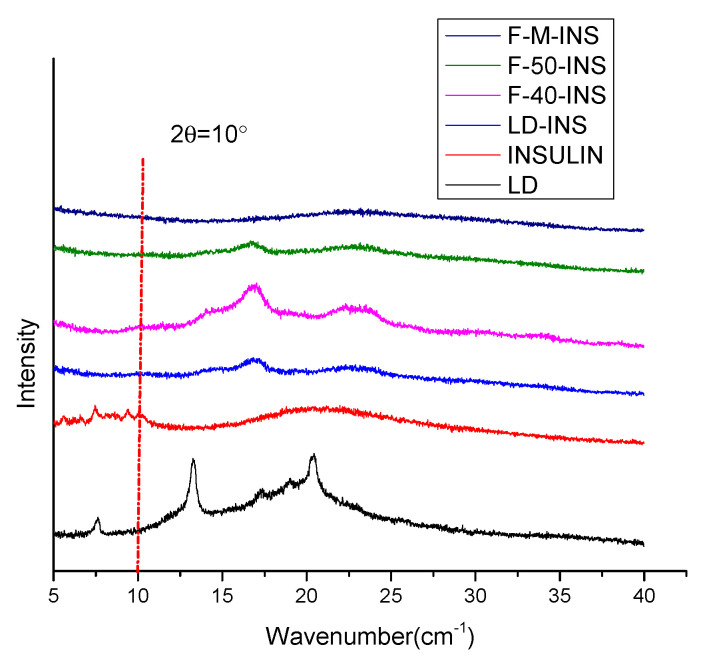
XRD patterns of LD, insulin, LD-INS, F-40-INS, F-50-INS, and F-M-INS inclusion complexes.

Figure 4 depicts the XRD spectra of LD, INSULIN, LD-INS, F-40-INS, F-50-INS, and F-M-INS. It can be seen that insulin showed crystalline peaks when 2θ was less than 10°, indicating that insulin is a mixture of amorphous and crystalline, which is consistent with the results reported by Zhang et al. [41]. In the spectrum of the binary nanoinclusion complexes, the crystal diffraction peaks of insulin almost completely disappeared, indicating that the insulin was dispersed in the linear dextrin in an amorphous state during the formation of the nanoinclusion complexes, which proved that the insulin was embedded in the linear dextrin.

It can be seen from the trace that the peak intensity and peak position changed greatly after adding insulin to the LD. This shows that there was an interaction between LD and insulin. We can see that the peak positions of the four complexes of LD-INS, F-40-INS, F-50-INS, and F-M-INS were basically unchanged, while the peak intensity showed a trend of first increasing and then decreasing, of which the diffraction peak of F-40-INS had the strongest peak intensity and the highest crystallinity. The peak intensity of the diffraction peak of F-M-INS was the lowest, and the peak position and intensity of the diffraction peak of LD-INS and F-50-INS were the closest.

### 3.6. CD Spectra

The secondary structure of a protein is defined by the patterns of hydrogen bonds between the backbone amino and carboxyl groups, which govern the conformation of the protein and its therapeutic functions [42,43]. The secondary structure of insulin was investigated using CD spectroscopy, which is regarded as one of the most effective methods to evaluate the secondary structure of proteins and peptides.

**Figure 5:**
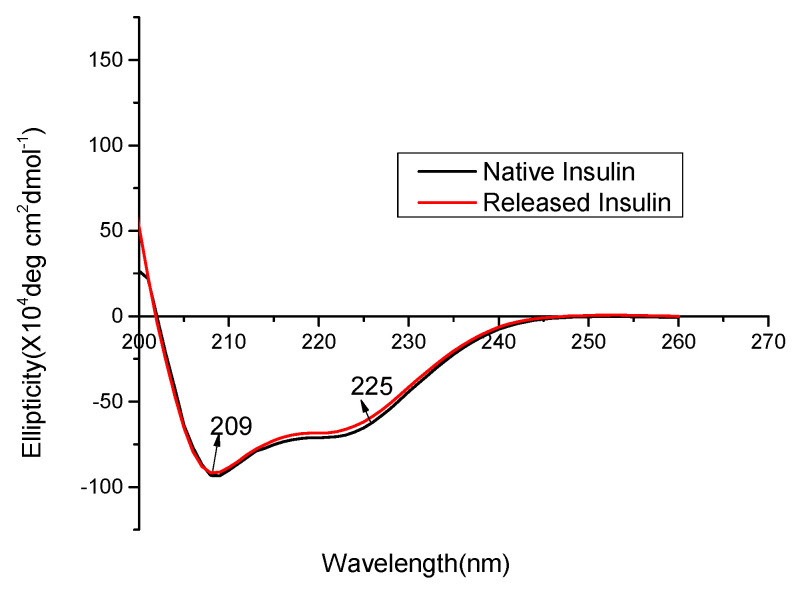
CD spectra of LD, insulin, LD-INS, F-40-INS, F-50-INS, and F-M-INS inclusion complexes.

Figure 5 shows the CD spectra of insulin in the native form (control group) and insulin released from LD-INS inclusion complexes. The CD spectra of the control insulin solution displayed two peak valleys at around 209 and 225 nm, which indicates the presence of a-helical structures in the insulin solution. This spectrum is also in accordance with the previously published results on the native insulin structure [44]. Importantly, insulin released from LD-INS inclusion complexes at the equivalent insulin concentration (0.1 mg/mL) also showed a similar pattern overlapping with that of the reference insulin solution. These results demonstrated negligible secondary structural changes of insulin. It was also reported in other studies that insulin preserved its secondary structure after being encapsulated into different nanoemulsions [45].

### 3.7. In Vitro Insulin Release

To predict the release profiles in conditions similar to the GI tract (stomach and intestine), the samples were first added to SGF at pH 1.2, which mimics the gastric environment. After 2 h, the release medium was changed to SIF, which simulates the transit to the intestinal environment. The pH of the GI tract increased gradually from gastric pH (1.2–2.5) to pH 5.0–6.0 in the small intestine and to 6.8 and 7.2 in the proximal and distal colonic regions, respectively [46].

In vitro release of insulin from LD-INS and F-M-INS inclusion complexes was investigated in pH 1.2 (the pH of gastric juice) and 6.8 (the pH of intestinal juice), and 7.4 PBS (the pH of blood plasma) at 37 °C.

**Figure 6:**
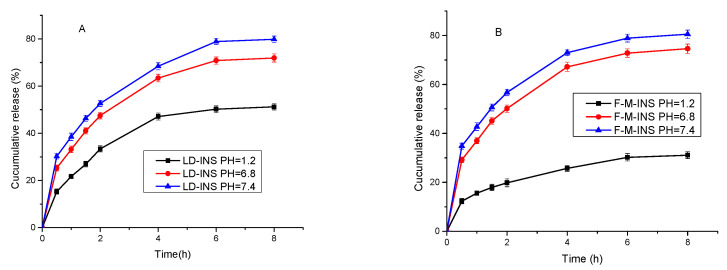
In vitro release of LD-INS (**A**) and F-M-INS (**B**) inclusion complexes.

It can be seen from Figure 6A,B that the insulin release of LD-INS and F-M-INS inclusion complexes was relatively slow under the condition of the simulated gastric juice pH 1.2, indicating that gastric juice has an inhibitory effect on the release of insulin. This may be due to the fact that insulin is positively charged at pH 1.2, and the isoelectric point of insulin is 5.3 [47]. Nonencapsulated insulin also precipitated upon the passage from pH 1.2 to pH 5.5. Insulin contains several functional groups with different charges and different acids or basic behaviors depending on the pH conditions. At pH 5.5 (approximately the insulin pI), the net charge of the amino acids was balanced, leading to a formally uncharged molecule, more non-polar, and with remarkably decreased solubility [48]. Insulin interacting with the negative charge of LD or F-M was attributed to the reduced release of insulin. The release rate at pH 7.4 and pH 6.8 was faster than that at pH 1.2. This is mainly because LD, F-M, and insulin are negatively charged under the pH conditions, and there is electrostatic repulsion between them, which is thus conducive to the release of insulin. It can be concluded that insulin is encapsulated in F-M; F-M-INS inclusion complexes showed a better effect on reducing the release of insulin in gastric juice and promoting the release of insulin in intestinal juice and blood plasma than LD-INS.

## 4. Conclusions

The reaction mainly involved hydrogen bonding and a hydrophobic interaction between LD and insulin in the process of inclusion complex formation. The optimal average DP of linear dextrin for encapsulating insulin was 16.10. The EE and LC of F-M-INS inclusion complexes reached their maximum values, which were 68.2% and 3.4%, respectively. F-M-INS inclusion complexes reduced the release of insulin under an acidic environment (pH 1.2) and promoted the release of insulin in the intestinal juice (pH 6.7) and blood plasma (pH 7.4). Moreover, insulin maintained its structure and was dispersed in linear dextrin in an amorphous state in the process of inclusion complex formation.

## Figures and Tables

**Table 1:** The relative molecular weight information of linear dextrin fractions.

	Mwp		Peak Areas (%)			Mwa		
Sample	Peak 1	Peak 2	Peak 1	Peak 2	Mn	Peak 1	PDI	Dp (Average)
LD	42706	1075	97.99	2.01	2233	3657	1.46	22.57
F-40	32475	1024	99.89	0.11	4280	5093	1.19	31.44
F-50	14550	N/A	100	N/A	2989	3538	1.18	21.84
F-M	9935	N/A	100	N/A	2333	2609	1.12	16.10

**Table 2:** Encapsulation efficiency and loading capacity of LD-INS, F-40-INS, F-50-INS, and F-M-INS inclusion complexes.

Sample	EE (%)	LC (%)
LD-INS	52.9 ± 0.9 ^d^	2.6 ± 0.1 ^b^
F-40-INS	41.6 ± 1.0 ^c^	2.2 ± 0.2 ^ab^
F-50-INS	48.7 ± 1.2 ^b^	2.8 ± 0.1 ^ab^
F-M-INS	68.2 ± 1.4 ^a^	3.4 ± 0.1 ^a^

Values with a different letter in the same column are significantly different (*p* < 0.05). Means in the same column that do not share a letter are significantly different.

## Data Availability

The data presented in this study are available on request from the corresponding author.
